# Burden and seasonality of RSV bronchiolitis in hospitalized children on a French Caribbean island: Practical lessons from a 13‐year study

**DOI:** 10.1002/jmv.70006

**Published:** 2024-11-11

**Authors:** Olivier Fléchelles, Camille Oger, Aude Charollais, Moustapha Drame, Rishika Banydeen, Fatiha Najioullah

**Affiliations:** ^1^ Paediatric and Neonatal Critical Care Service, CHU Martinique (University Hospital of Martinique) Fort‐de‐France France; ^2^ Univ Montpellier, INSERM, EFS, Univ Antilles Montpellier France; ^3^ CHU Martinique, Service de Pédiatrie Fort de France France; ^4^ CHU Martinique, Unité de Soutien et Méthodologie de la Recherche Fort‐de‐France France

**Keywords:** bronchiolitis, epidemiology, infants, RSV epidemics, seasonal incidence, tropical climate

## Abstract

Epidemiology of respiratory syncytial virus (RSV) bronchiolitis in the tropics is poorly understood, complicating the effective management of RSV epidemics. The main objective was to describe the seasonality of RSV bronchiolitis epidemics and the clinical characteristics of hospitalized infants over a 13‐year period on the French Caribbean island of Martinique. Single‐center retrospective observational study including infants under 2 years of age hospitalized at the Martinique University Hospital for RSV‐positive bronchiolitis from January 2007 to December 2019. One thousand two hundred thirty‐tree cases were included. Epidemics occurred during the rainy season, beginning in September, peaking in October/November and ending in December. A distinct biennial seasonality pattern was observed, with alternating years of high and low incidence. Mean duration of epidemics was of 11 weeks. Clinical characteristics of patients were similar to those hospitalized in temperate areas. Median hospital stay was 4 days. Median age was 3 months, 14.1% of patients were born prematurely, 2.5% had congenital heart disease and 41.1% required oxygen therapy. In Martinique, RSV bronchiolitis epidemics in infants occur in a regular biennial pattern during the rainy season. An accurate knowledge of the local seasonality will allow us to better anticipate hospital organization before epidemics.

## 1 INTRODUCTION

Respiratory syncytial virus (RSV) infections are the leading cause of hospitalization for respiratory distress in young children, with nearly all children infected by the time they reach 2 years of age.^1^ RSV infections can be very severe, and account for 66,000 to 199,000 deaths in children under 5 years worldwide.^2^ RSV is a highly contagious virus, with both airborne and hand‐borne transmission that thrives in damp conditions.^3^

In temperate regions where this infection has been well studied, it occurs preferentially from November to March, probably favored by the cold and humid conditions, as well as by the confinement of individuals during this season. The severity of the disease is greater if the child is young, premature, or has a chronic respiratory disease or congenital heart disease.^4^ In the absence of an effective vaccine for young children, prevention is based on standard hygiene measures and injection of monoclonal antibodies (palivizumab or the new antibody, nirsevimab) before the start of the epidemic season to be effective.^5^, ^6^, ^7^ Hence, it is important that the start of epidemic season be precisely known for each world area. This is however not the case in the tropics, where little research has been conducted to date. Some epidemiological studies carried out in tropical countries have shown that viral circulation exists throughout the year, with higher intensity during the rainy season, which is consistent with the preference of RSV for humidity.^8^ Accordingly, in Puerto Rico, a 4‐year study showed hospitalizations throughout the year, with epidemics occurring during the period of maximum humidity, i.e. from August to November.^9^ The same pattern was observed in the English and Dutch speaking Caribbean islands, with RSV epidemics mainly occurring from May to October, or in Trinidad from June to December.^10^, ^11^ The humidity level, the number of rainy days, the amount of sunshine and cloud cover, as well as wind strength, could explain the variations in seasonality in tropical areas. Seasonality is related to virus circulation patterns and is defined as the ability to reappear at the same time from 1 year to the next. The epidemiology of RSV in Martinique, a small French island in the Caribbean basin with a typically tropical climate, has previously been studied over a 2 year period, but the observation period was not long enough to ensure that the seasonality observed would be systematically repeated from 1 year to the next.^12^ In addition, that study did not describe the number and clinical characteristics of affected children hospitalized on the island.

The primary objective of the present study was to report the characteristics of RSV bronchiolitis epidemics (seasonality and incidence rate) in hospitalized infants in Martinique over a 13‐year period (2007–2019). Secondarily the clinical characteristics of the hospitalized infants over the study period were described and the potential factors associated with disease severity were analyzed.

## 2 METHODS

### 2.1 Study context and design

Martinique is a French overseas territory in the Caribbean with a health care system identical to that of mainland France. It has a tropical climate, alternating between a dry season from January to June, and a rainy season from July to December. As of the January 1 2018, the population of Martinique was amounted to 368,783 inhabitants, with 3670 live births registered in 2018. Martinique has a single University hospital which receives all pediatric hospitalizations on the island.

This single‐center retrospective observational study that included all infants under 2 years of age hospitalized for RSV bronchiolitis was conducted at the University Hospital from January 1 2007 to December 31 2019. The following exclusion criteria were applied: children aged two and above at the time of hospitalization, lack of hospitalization, children in foster care, children who had already died at the time of data collection, parental oppositions, incorrect mailing address and duplicate patient records.

All hospitalized children initially transited via the Pediatric Emergency Department. The diagnosis of bronchiolitis was made clinically at admission by the attending pediatrician based on the National Institute for Health and Care Excellence definition (NICE), namely a child presenting with rhinitis lasting 1–3 days, followed by persistent cough AND either tachypnea or chest recession (or both) AND either wheezing or crackles (or both) on chest auscultation.^13^ The diagnosis of RSV infection was confirmed by the positivity of a respiratory sample (nasopharyngeal swab, nasal washes or nasopharyngeal aspirate) from each hospitalized child, prospectively tested by immunofluorescence from January 1 2007 to January 31 2016, and then by real‐time RT‐ PCR from February 1 2016 onwards.

For immunofluorescence testing two kits (MonoFluoTMScreen RSV Biorad, France) were used from 2007 to 2008, and afterwards « Antivirus Respiratoire syncytial conjugué à la fluorescéine » (Argène/Biomérieux, France) from 2009 to January 2016. Nucleic acid detection, extraction and purification of RNA was carried out on NucliSens EasyMag (BioMérieux) and amplification with « RSV/hMPV r‐gene® » kit (Argène/Biomérieux) on an *Applied Biosystems*™ *7500* Real‐Time PCR Systems (Thermo Fisher Scientific). The quality control of virus detection was assessed: (1) for immunofluorescence: by using positive and negative slides (Argène/Biomérieux, France) and in house slides made with samples from previous patients; (2) for real time RT‐PCR: the University Hospital's virology laboratory participates every year in the annual program for External Quality Assessment (EQA)/Proficiency Testing (PT) from Quality Control for Molecular Diagnostics (QCMD) (Scotland, UK). For extraction/amplification of RNA, negative and positive controls (strain dilutions) were included in every round. Differentiation between RSVA and RSVB was performed retrospectively, by real time RT‐PCR on 588 aliquots stored at −80°C, from randomized samples, using the Seegene Allplex Respiratory Panel one kit (Eurobio).

### 2.2 Study data

#### 2.2.1 Clinical data

For each child the following items were recorded, the date of diagnosis, age and sex, length of hospital stay, admission status to the pediatric intensive care unit (PICU), need for supplemental oxygen therapy, enteral nutrition, respiratory assistance (Noninvasive support, invasive ventilation), presence of risk factors such as prematurity (babies born before the 37th week of gestation), bronchopulmonary dysplasia, congenital heart disease, nosocomial bronchiolitis, any hospital readmission due to recurrence of RSV infection before the age of 2 years, and death during hospitalization. All data were retrospectively retrieved from medical records.

Noninvasive respiratory support comprised high flow nasal cannula (HFNC) and noninvasive ventilation. Nosocomial bronchiolitis was defined as bronchiolitis that occurred secondarily while the patient was initially hospitalized for another reason, and RSV was isolated at least 48 h after admission. Readmission was defined as a second hospitalization for RSV bronchiolitis before the age of 2 years.

#### 2.2.2 Disease seasonality

Epidemic features were defined as follows^8^, ^14^: (1) Pre‐epidemic phase: first week with three positive RSV cases; (2) Epidemic threshold: five or more weekly RSV positive cases; (3) Epidemic onset: at least 5 weekly cases for two consecutive weeks, with the first week chosen as the threshold date; (4) End of the epidemic: if there are fewer than five cases for two consecutive weeks, the second week is considered as the last week of the epidemic; (5) Peak of the epidemic: week with the maximum number of positive cases; (6) Duration of the epidemic: difference between the beginning and the end of the epidemic, in weeks.

### 2.3 Statistical analysis

For all descriptive and inferential analyses, normality of the data distribution was tested. Means and standard deviations were reported for normally distributed variables, and medians and interquartile ranges (IQR) for non‐normally distributed variables. Categorical variables were reported as numbers and percentages. The following tests were used for group comparisons when appropriate: Student t test, Wilcoxon‐Mann‐Whitney test, Chi square test and Fisher's exact test. To investigate the factors potentially associated with disease severity (defined as hospitalization in a PICU or the use of supportive techniques (oxygen therapy, feeding tube, respiratory support)), univariable and multivariable logistic regression models were implemented. Variables with either a *p*‐value < 0.25 in univariate analysis, or of clinical interest, were considered for multivariable logistic regression. Backward stepwise selection was used to fit the final model. Associations were reported as odds ratios (OR) and 95% confidence intervals (CI). All statistical analyses were performed using Statistical Analysis System (SAS) version 9·4 (SAS Institute Inc.). The level of statistical significance was set at *p* < 0.05.

## 3 RESULTS

**Figure 1:**
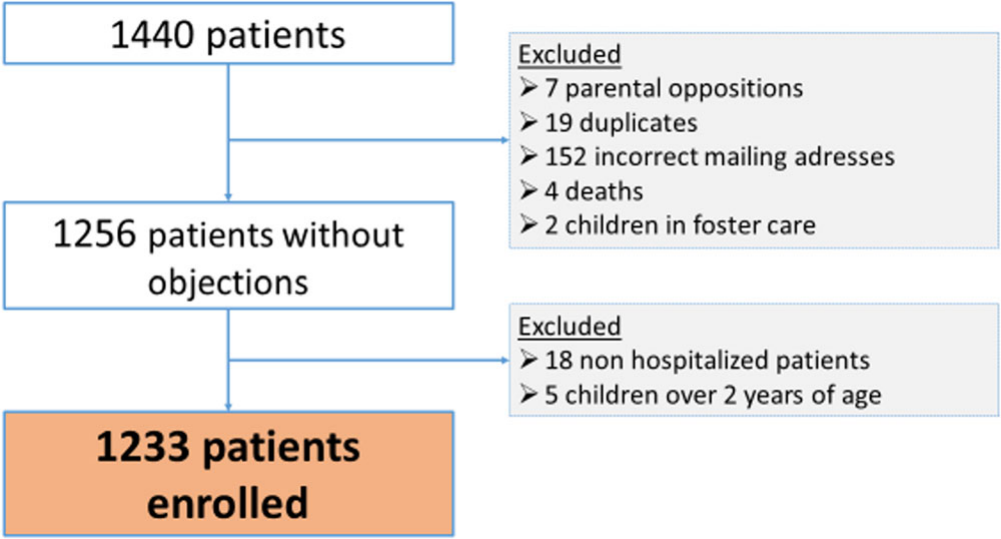
Flow‐chart, January 2007 to December 2019. Missing data for serotypes in 2011, 2012 and 2013.

A total of 1440 patients hospitalized for bronchiolitis were initially identified as RSV positive, of whom 1233 (85.6%) were finally included for study purposes (Figure 1).

### 3.1 Seasonality and characteristics of RSV epidemics

**Figure 2:**
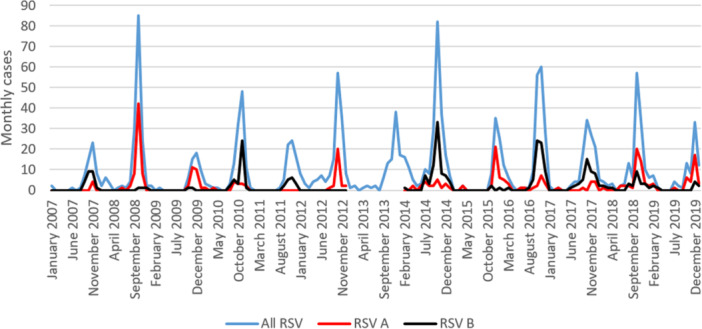
Monthly distribution of hospitalized cases of RSV bronchiolitis (RSV A, RSV B, and RSV A + B) in infants under two years in Martinique, 2007–2019. It is a high incidence year if the number of monthly cases exceeds 35.5. It is a low incidence year if the annual number of cases is less than 35.5. High‐incidence years are shown in red, low‐incidence years in blue. The lines or dashes identify the different years among those of low or high incidence. RSV, respiratory syncytial virus.

**Figure 3:**
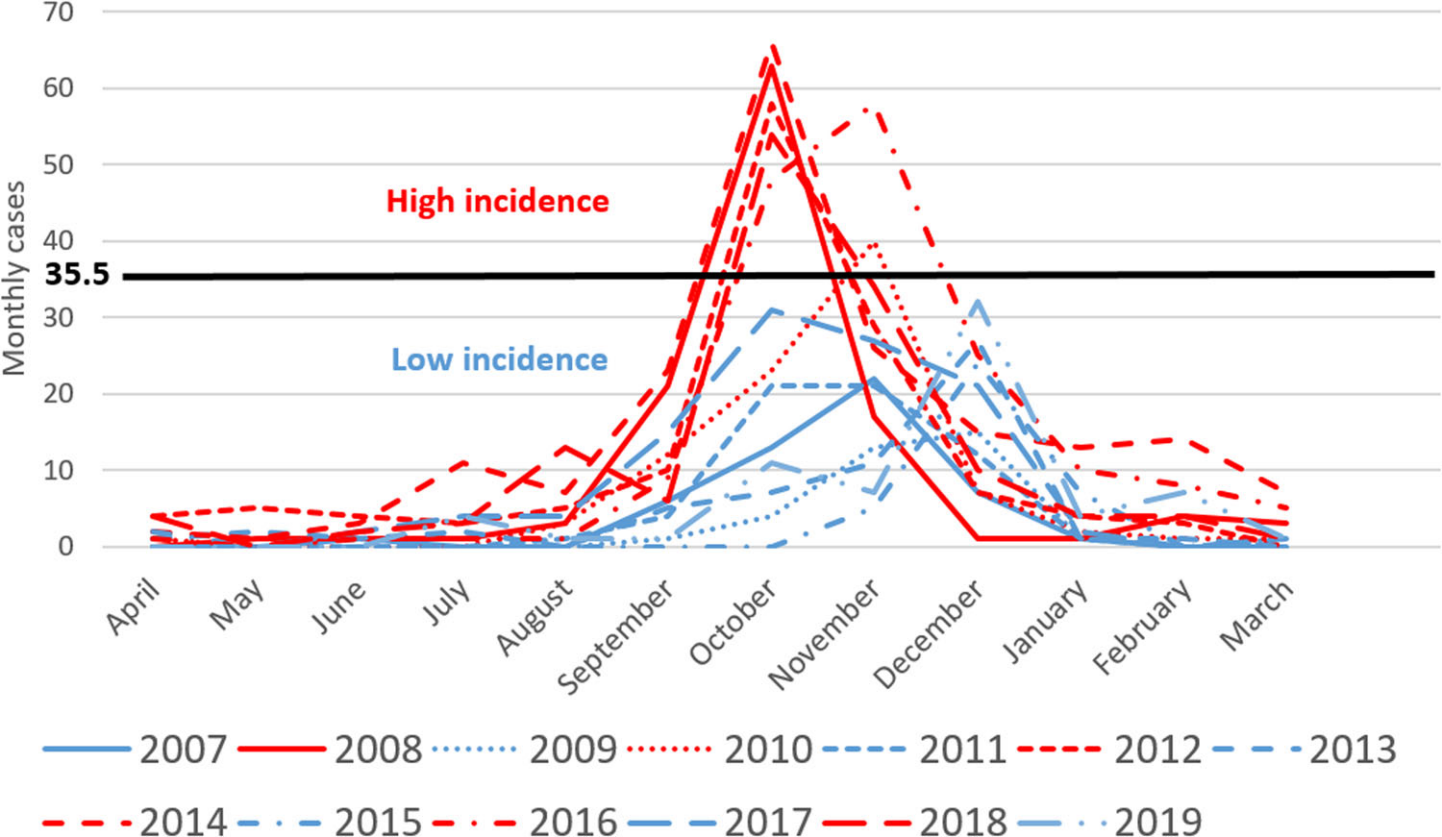
Monthly distribution of hospitalized cases of RSV bronchiolitis by year in infants under two years in Martinique, 2007–2019. RSV, respiratory syncytial virus.

Hospitalizations followed a biennial pattern, with alternating years of high and low incidences (*p* = 0.008) (Figure 2 & 3). The terms “high incidence” and “low incidence” were used when the incidence was above or below the mean of the maximal number of monthly cases (35.5), respectively (Figure 3). The median annual cases varies by a factor of 2.3 between high incidence and low incidence years (Table 1). However, it can vary by as much as a factor of 4.4 if we compare the lower and upper limits of the interquartile ranges between consecutive high incidence and low incidence years. A pre‐epidemic phase occurred 3 weeks before the onset of the epidemic (Table 1). Epidemics generally started between mid‐September and late September, with the peak mainly observed in October/November, and the end in December. Epidemic peak was reached in three to 4 weeks regardless of incidence. The main differences from low‐incidence years (apart from the incidence itself) were: more dispersed epidemics, a much lower number of children hospitalized per week at peak (eight cases vs. 19 cases), and more than half the number of cases during the interepidemic period (median of 4.06 cases per month vs. 9.96 cases per month in high‐incidence years). On the other hand, the duration of the epidemic did not differ between high and low incidence, with a median duration varying between 9 and 11 weeks (*p* = 0.78).

**Table 1:** Epidemiological characteristics according to incidence level, of hospitalized cases of RSV bronchiolitis in infants under two years in Martinique from 2007 to 2019.

Epidemiological characteristics	Low incidence (m; IQR)	High incidence (m; IQR)	P value
Duration of the outbreak (w)	9 (7–13)	11 (11–12)	0.78
Number of weeks before epidemic	3 (1–4)	3 (2–8)	0.77
Number of weeks between beginning and peak of epidemic	4 (2–8)	3 (3–4)	0.41
Number of cases per outbreak phase	37 (29–56)	97.5 (91–125)	0.02
Number of weekly cases at peak	8 (7–10)	19 (15–22)	>0.001
Number of monthly cases in interepidemic phase	2 (1–7)	15 (10–19)	0.02
Number of annual cases	58 (38–66)	131.5 (116–167)	0.02

Abbreviations: m, median; IQR, interquartile range; RSV, respiratory syncytial virus; w, weeks.

RSV genotyping could be performed on 48% of all specimens over the 13‐year study period. It should be noted that due to the loss of stored samples (a freezer broke down), no genotyping could be performed on the 2013 samples and only on 23.3% and 20.6% of the 2011 and 2012 samples, respectively. Although both genotypes were in circulation during each epidemic, one genotype (A or B) was always predominant, depending on the year (Figure 2). Genotype (A or B) was not associated with incidence level (high or low incidence year) (*p* = 1.00).

### 3.2 Patients' clinical characteristics

Among the 1233 patients, there were more boys than girls (56.1% vs. 43.9%), and median age was 3.2 months (Table 2). The median length of hospital stay was 5 days for all wards, and 8 days for PICU. Regarding severity criteria, 14.2% were former premature infants, 2.4% had congenital heart disease and 1.7% had bronchopulmonary dysplasia. In total, 282/1233 patients (22.9%) were admitted to PICU. Among the latter, 203 (72%) were 3 months old or younger, 61 (21.6%) were born prematurely, 13 (4.6%) had congenital heart disease and nine (3.2%) had bronchopulmonary dysplasia. Genotype A or B was not associated with the severity of bronchiolitis (*p* = 0.74).

**Table 2:** Characteristics and clinical severity criteria of hospitalized cases of RSV bronchiolitis in infants under two years in Martinique from 2007 to 2019 (*N* = 1233).

Characteristics	Parameters
Age, months	3.2 (1.6–6.6)
Length of hospital stay, days^*n* = 1230^	5.0 (4.0–8.0)
Sex	
‐Boys	692 (56.1)
‐Girls	541 (43.9)
Hospitalization in PICU^*n* = 1220^	282 (22.9)
Enteral nutrition^*n* = 1215^	475 (38.5)
Oxygen therapy^*n* = 1214^	502 (40.7)
Ventilatory support^*n* = 1210^	237 (19.4)
Non invasive support	213 (17.6)
Invasive ventilation	44 (3.6)
History of preterm birth^*n* = 1214^	172 (14.2)
History of bronchopulmonary dysplasia^*n* = 1212^	21 (1.7)
History of congenital heart disease^*n* = 1214^	29 (2.4)
Readmission^*n* = 1214^	85 (7.0)
Nosocomial bronchiolitis^*n* = 1213^	81 (6.7)
Death^*n* = 1214^	1 (0.1)

*Note*: Quantitative variables are presented as median (interquartile range, IQR), and qualitative variables as absolute values (percentages).

Abbreviations: IQR, interquartile range; PICU, Pediatric Intensive Care Unit; RSV, respiratory syncytial virus.

Multivariable logistic regression analysis underlined significantly higher risks (*p* < 0.05) for admission to in the presence of the following severity criteria: history of prematurity (OR (95%CI): 2.2(1.5–3.2), history of congenital heart disease (OR (95%CI): 2.3(1.0–5.4), history of bronchopulmonary dysplasia (OR (95%CI): 3.4 (1.3–8.8), age less than 3 months (OR (95%CI): 4.2(3.1–5.7). Prematurity and an age under 3 months were also independent predictors for the use of support techniques (oxygen therapy, feeding tube, ventilatory support) (*p* < 0.05) (Table 3).

**Table 3:** Clinical severity according to selected characteristics of hospitalized cases of RSV bronchiolitis in infant under two years in Martinique from 2007 to 2019 (*N* = 1233): multivariable logistic regression analysis.

Severity markers (response variables)	Patient characteristics (explanatory predictors)			
	Preterm	Heart disease	BPD	Age < 3 months
	OR (95% CI)	OR (95% CI)	OR (95% CI)	OR (95% CI)
Admission to PICU	2.22 (1.52–3.24)	2.34 (1.02–5.37)	3.42 (1.32–8.84)	4.20 (3.10–5.70)
Use of support techniques				
Oxygen therapy	1.95 (1.38–2.75)	1.73 (0.78–3.85)	1.01 (0.39–2.57)	3.12 (2.45–3.98)
Feeding tube	1.87 (1.32–2.65)	2.68 (1.18–6.11)	1.09 (0.42–2.82)	3.55 (2.77–4.54)
Ventilatory support	2.53 (1.71–3.74)	3.51 (1.52–8.12)	1.47 (0.48–4.51)	4.47 (3.20–6.24)

Abbreviations: BPD, history of bronchopulmonary dysplasia; CI, Confidence Interval; heart disease, history of congenital heart disease; OR, Odds‐Ratio; PICU, Pediatric Intensive Care Unit; preterm, history of preterm birth; ventilatory support, high flow nasal cannula (HFNC), noninvasive ventilation, invasive ventilation.

## 4 DISCUSSION

The epidemiology of bronchiolitis in the tropics is poorly documented, and this study in a typical Caribbean Island, over a long time period of 13 years, provides several useful insights. In Martinique, RSV bronchiolitis epidemics evolve in a biennial pattern, alternating between years of high incidence and years of low incidence (Figure 2). It should be noted that viral circulation is possible even during the dry season, and that it occurs mainly during the few months preceding the years of strong epidemic waves. The epidemic peak generally occurs between the months of October and November, especially in years of high incidence, contrary to what was reported in a study by Bloom‐Feshbach et al. investigating geographic variations in seasonality in 137 global locations.^14^ The Bloom‐Feshbach team described a monthly peak between May and July in the tropical regions of the Northern Hemisphere, but with a very scattered distribution.^14^ In Martinique, two distinct phases of epidemic onset were identified: a pre‐epidemic phase and an ascending phase between the beginning of the epidemic and its peak. The duration of each phase was about 4 weeks, and varied little from year to year. Each epidemic usually lasted about 3 months, regardless of the number of incident cases (Figure 3). It should be noted that the number of incident cases varied greatly from year to year, as the observed variation was by a factor of 4·4.

This biennial variation in RSV circulation has been widely described in the literature and has multifactorial causes. A recent multicenter study conducted from 2001 to 2017, found the same year to year variability in Finland, Norway and Denmark, but it was not observed in Italy, England or Scotland.^15^ These observed changes are not related to changes in the predominance of RSV A and B genotypes, although this has been documented elsewhere.^14^ The development of tourism, with a large influx of people from mainland France during the dry season (January–June), does not seem to explain the onset of the RSV epidemic in Martinique, as it begins about 2 months before the epidemic period in France.^16^ Barrier measures, which were only marginally applied before the COVID‐19 epidemic in Martinique (March 2020 to November 2021), also do not seem to explain these annual variations. It seems that the biennial pattern observed in Martinique is probably related to herd immunity after infection.^17^, ^18^ In Martinique, as in many tropical regions, epidemics occur during the hottest and wettest months of the year. This is not surprising, since RSV is known to thrive in wet, humid conditions.^19^ However, these associations are not found to be significant in all studies, suggesting that many other environmental factors are at play and can potentially modify the pattern of epidemics.^20^, ^21^ Air pollutants, such as ozone and traffic pollutants, have been associated with an exacerbation of respiratory infections in children under 5 years of age.^22^ This has also been described in Martinique, which is regularly impacted by natural pollution from the Sahara desert, known as “sand haze.”^23^ In combination with other meteorological phenomena, the latter could be a factor in the variation of RSV epidemics among children in Martinique.

The population of RSV‐infected infants in this study is similar to that of temperate populations. However, a higher proportion of premature infants hospitalized for bronchiolitis was observed. In mainland France, a multicenter study in 2015 showed that only 3.3% of all patients hospitalized for bronchiolitis were born prematurely, compared to 14.0% in this study (Table 2).^24^ A study in Spain from 1997 to 2011 among infants under 2 years of age described similar rates of comorbidities in terms of heart disease (2.5%) and bronchopulmonary dysplasia (1.1%). However, only 0.4% were born preterm in the Spanish study.^25^ This difference can be explained by a prematurity rate that is 1.5 times higher in Martinique than in mainland France (10.1% vs. 6.6% respectively), although other unidentified confounders are probably involved.

During the study period, 282 patients were admitted to the PICU in Martinique, i.e. a rate of 22.9%, which is quite similar to studies conducted in mainland France (Table 2).^26^ The use of assistive devices is very similar to that described in other regions of the world.^2^, ^27^, ^28^ The results are therefore generally quite similar to those described in the literature. Prematurity, an age under 3 months, congenital heart disease and chronic respiratory disease are well described factors of disease severity, and are widely represented in this population. However, it is to be considered even more at risk because of the high rate of prematurity. It is therefore important to intensify prevention and in particular, to insist on the discharge of prematurely‐born infants from neonatology units during RSV epidemic periods. In the present study, it can be noted a congruent rate of nosocomial bronchiolitis, which underline a need for intensified preventive efforts among caregivers.^29^, ^30^

Furthermore, the observations from this study are very important from a practical point of view, as the study findings will be useful in predicting whether the next epidemic will be strong or weak, thanks to two complementary signals. The first signal is the alternation between low and high incidence levels. The second signal is the existence of viral circulation during the dry season, with hospitalization of infants for RSV bronchiolitis even outside of the period favorable to its development, i.e. during the months preceding a strong epidemic. This predictive capability will enable us to enhance epidemic preparedness in the best possible way, by ensuring the presence and functionality of all the necessary equipment to face the epidemic. Advanced knowledge of the likely onset of an epidemic could enable us to adjust medical and paramedical staff resources to ensure availability in large numbers during the epidemic. It will also be easier to anticipate information campaigns and alerts on the occurrence of bronchiolitis and the need to apply barrier measures. In addition, appropriate measures must be taken to ensure that all newborns and all eligible children receive serotherapy (the new nirsevimab or the older palivizumab) between mid‐July and the end of July, which is two to 3 months earlier than practices in temperate zones. Finally, thanks to this ability to detect the pre‐epidemic phase, the imminent arrival of the epidemic can be predicted. This will enable us to prepare for the influx of patients by organizing units into bronchiolitis and non‐bronchiolitis sectors to limit nosocomial risks, as well as providing training and frequent reminders to caregivers about hygiene precautions.

To the best of our knowledge, this is the first study in the French West Indies and the Caribbean to investigate the seasonality of bronchiolitis, and the severity criteria over a long period of time with a large sample of patients and a low rate of missing data. These data are robust and enable us to better understand and anticipate future epidemics. The combination of hospital and sentinel network data, as well as the results of this study, could enable health authorities to improve surveillance and remain vigilant about the risk of occurrence and follow‐up of an ongoing epidemic. These novel data raise the perspective of an alert threshold for vigilance regarding the occurrence of a large‐scale epidemic, on top of the surveillance already carried out according to international recommendations.^31^

Clinical data were based on hospital informatics data and chart review. These data are available at the institutional level, and have previously been validated for the description of bronchiolitis epidemics. However, because they are based on diagnostic coding, there might be potential measurement bias. However, the very low rate of missing data and the simplicity of the search criteria probably greatly reduced this bias. It should also be noted that the study population used a virological diagnosis to identify infected patients. Virological investigation in infants is systematically associated with the confirmation of clinical acute bronchiolitis, the diagnosis of which is simple and well codified. Therefore, the number of patients who escaped screening is probably extremely low.^13^

## 5 CONCLUSION

Epidemics of RSV bronchiolitis in infants under 2 years in Martinique occur during the rainy season and follow a regular biennial pattern, with years of high incidence alternating with years of low incidence. In years of high incidence, RSV circulates even during the dry season preceding the epidemic, which is a relevant indicator. The epidemic usually begins between the first half of September and the second half of October and ends between the last week of November and the second half of January of the next year. The clinical characteristics of infants in Martinique are similar to those described in temperate and other tropical regions.

By describing the seasonality and characteristics of RSV epidemics over 13 years, these findings clarify the dynamics and intensity of this pathology in the infant population of Martinique. These new data will enable us to adapt preventive and prophylactic measures to better protect Martinique's infants. However, variations in incidence likely depend on many other unmeasured parameters, such as the collective immunity in the population, weather conditions or air pollution. All these elements warrant further investigation in future studies, to enhance knowledge of the RSV virus, and to better adapt epidemic preparedness and response.

## AUTHOR CONTRIBUTIONS

**Olivier Fléchelles**: Conceptualisation; formal analysis; writing. **Camille Oger**: Investigation; original draft preparation. **Aude Charollais**: Critical review. **Moustapha Drame**: Methodology; validation; critical review. **Rishika Banydeen**: Methodology; validation; data curation; formal analysis; critical review. **Fatiha Najioullah**: Conceptualisation; formal analysis; critical review. All authors have read and agreed to the published version of the manuscript.

## CONFLICT OF INTEREST STATEMENT

The authors declare no conflict of interest.

## ETHICS STATEMENT

Each child's legal representative was informed about the study by personal letter, giving them the opportunity to explicitly refuse the use of their child's medical data for research purposes, in accordance with French legislation. The absence of explicit opposition from the legal representative was documented in each patient's medical record. The study was approved by the Institutional Review Board (IRB) of the University Hospital of Martinique (on 19 June 2019 under the reference number 2019/009).

## CLINICAL TRIAL REGISTRATION

NCT04415229.

## Data Availability

Data will be made available upon reasonable request to Dr Olivier Fléchelles (corresponding author; olivier.flechelles@chu-martinique.fr).
